# Circulating neutrophils activated by cancer cells and M2 macrophages promote gastric cancer progression during PD-1 antibody-based immunotherapy

**DOI:** 10.3389/fmolb.2023.1081762

**Published:** 2023-06-01

**Authors:** Chenfei Zhou, Liting Guo, Qu Cai, Wenqi Xi, Fei Yuan, Huan Zhang, Chao Yan, Lei Huang, Zhenggang Zhu, Jun Zhang

**Affiliations:** ^1^ Department of Oncology, Ruijin Hospital, Shanghai Jiao Tong University School of Medicine, Shanghai, China; ^2^ Department of Oncology, Wuxi Branch of Ruijin Hospital, Shanghai Jiao Tong University School of Medicine, Wuxi, China; ^3^ Department of Pathology, Ruijin Hospital, Shanghai Jiao Tong University School of Medicine, Shanghai, China; ^4^ Department of Radiology, Ruijin Hospital, Shanghai Jiao Tong University School of Medicine, Shanghai, China; ^5^ Department of Gastrointestinal Surgery, Ruijin Hospital, Shanghai Jiao Tong University School of Medicine, Shanghai, China

**Keywords:** gastric cancer, neutrophils, immune checkpoint inhibitors, tumor microenvironment, PD-1

## Abstract

**Aims:** To analyze the correlation between the neutrophil-to-lymphocyte ratio (NLR) and prognosis of advanced gastric cancer (AGC) patients treated by PD-1 antibody-based therapy and to delineate molecular characteristics of circulating neutrophils by single-cell RNA sequencing (scRNA-seq).

**Methods:** The clinicopathological information of 45 AGC patients receiving PD-1 antibody-based regimens at the Department of Oncology, Ruijin Hospital, was reviewed. Treatment outcomes including objective response rate (ORR), progression-free survival (PFS), and overall survival (OS) were recorded. The correlation between NLR and efficacy of PD-1 antibody-based treatment was analyzed. Single-cell RNA sequencing (scRNA-seq) analysis was performed based on multisite biopsy samples from two AGC patients to explore the molecular characteristics of circulating neutrophils and their pro-tumor mechanisms. Tissue samples from 88 gastric cancer patients who underwent radial gastrectomy were collected for immunochemistry staining.

**Results:** A high posttreatment NLR was associated with poor outcomes of AGC patients receiving PD-1 antibody-based regimens. scRNA-seq analysis showed that an increased number of circulating neutrophils were found in peripheral blood samples after treatment in which neutrophil cluster 1 (NE-1) was the major subcluster. NE-1 was featured with a neutrophil activation phenotype with the high expression of *MMP9*, *S100A8*, *S100A9*, *PORK2*, and *TGF-β1.* NE-1 displayed an intermediate state in pseudotime trajectory analysis with gene function enrichment found in neutrophil activation, leukocyte chemotaxis, and negative regulation of MAP kinase activity. Cellular interaction analysis showed that the chemokine signaling pathway is the major interactional pathway of NE-1 between subclusters of malignant epithelial cells (EP-4) and M2 macrophages (M2-1 and M2-2). In turn, the MAPK signaling pathway and Jak-STAT signaling pathway of EP-4, including IL1B/IL1RAP, OSM/OSMR, and TGFB1/TGFBR2 axes, were identified as interacting pathways between EP-4 and NE-1. The high expression of OSMR in tumor cells was closely correlated with lymph node metastasis of gastric cancer.

**Conclusion:** The posttreatment NLR could be a poor prognostic marker of AGC patients treated with immune checkpoint inhibitors (ICIs). Subclusters of circulating neutrophils activated by tumor cells and M2 macrophages could participate in gastric cancer progression through signaling interactions with tumor cells.

## Introduction

Advanced gastric cancer (AGC) is one of the most malignant diseases worldwide. The median overall survival of AGC patients is barely over 12 months (Cunningham et al., 2008; Gao and Wu, 2019; Wang et al., 2019). With the development of immune checkpoint inhibitors (ICIs), the efficacy of PD-1 antibodies has been demonstrated in AGC patients (Sharma et al., 2021). ATTRACTION-2 and REGONIVO trials showed the efficacy of nivolumab and nivolumab plus regorafenib in chemotherapy-refractory late-stage AGC patients, respectively (Kang et al., 2017; Fukuoka et al., 2020). Recently, CheckMate-649 and ORIENT-16 trials demonstrated the superior efficacy of PD-1 inhibitors plus chemotherapy by comparing with chemotherapy alone as the first-line therapy for HER2-negative AGC patients (Xu et al., 2019; Janjigian et al., 2021a). For HER2-positive AGC patients, the results of KEYNOTE-811 trials showed that adding pembrolizumab to chemotherapy plus trastuzumab significantly improved the objective response rate (Janjigian et al., 2021b).

Currently, PD-1 antibodies have been recommended in clinical practices to treat AGC patients based on these evidence reports, although some patients still cannot benefit from PD-1 antibody-based therapy and even suffer a rapid disease progression (Kundel et al., 2020). The objective response rate (ORR) of PD-1 antibody monotherapy in late-stage and treatment-refractory AGC patients is only approximately 10%–20% (Kang et al., 2017). Biomarker investigations of ICIs have been performed aiming to identify patients who are more likely to respond to the treatment (Bai et al., 2020). For gastric cancer patients, a combined positive score (CPS) based on PD-L1 expression, microsatellite status, and Epstein–Barr virus status are now recommended to be detected before ICI treatment.

The neutrophil-to-lymphocyte ratio (NLR), which is a mini-invasive, low-cost, and real-time method, is now identified as a surrogate biomarker correlating with the outcome of ICI treatment and has been reported in several kinds of tumors, including lung cancer, renal cancer, and melanoma (Ocana et al., 2017). A high NLR before or during treatment indicated a poorer outcome of cancer patients treated with ICIs (Valero et al., 2021). Gou et al. (2021) reported that a pretreatment-elevated NLR was significantly associated with inferior PFS and OS in AGC patients receiving anti-PD-1 inhibitors. However, these previous studies were mainly focused on correlation analysis. The underlying molecular mechanisms of elevated circulating neutrophils in promoting cancer progression during ICI treatment are still under investigation.

Biological functions and molecular features of tumor-infiltrating immune cells including tumor-associated neutrophils (TANs) are investigated, and a sophisticated interaction between tumor cells and host microenvironment has been recognized (Hanahan, 2022). Tumor-associated neutrophils play important roles in regulating tumor angiogenesis, extracellular matrix remodeling, metastasis, and immunosuppression (Jaillon et al., 2020). The correlation between elevated circulating neutrophils and patient outcomes suggested that neutrophils in peripheral blood may also possess pro-tumor activity, which has not been fully explored in gastric cancer.

In the present study, we aimed to further investigate the prognostic value of the NLR level and delineate the molecular characteristics of circulating neutrophils by single-cell RNA sequencing (scRNA-seq) in AGC patients receiving PD-1 antibody-based regimens.

## Materials and methods

### Patients and samples

The clinicopathological information of 45 gastric cancer patients who underwent systemic therapy from July 2020 to December 2021 at the Department of Oncology, Ruijin Hospital was collected retrospectively. All patients were pathologically confirmed as having gastric adenocarcinoma with distant metastases treated with PD-1 antibody-based therapy. The treatment results including objective response rate (ORR) and progression-free survival (PFS) were recorded. The NLR at the time of baseline (T0), before the second cycle (T1), and first imaging assessment of efficacy (usually at 9 weeks, T2) was calculated based on the results of patients’ routine blood tests.

For scRNA-seq, multisite biopsy samples from patient 1 (P1) and patient 2 (P2) in this cohort were obtained before and after treatment, including samples from stomach tumor (ST), peripheral blood (PB) and pleural fluid (PL), and ascites (AS). Written informed consent was provided before biopsy. Paraffin-embedded specimens from 88 gastric cancer patients who underwent radial gastrectomy were collected. All samples were pathologically confirmed as gastric adenocarcinoma. Clinicopathological data were reviewed and are listed in Supplementary Table S1. The protocol was approved by the Ethics Committee of Ruijin Hospital, Shanghai Jiao Tong University School of Medicine, Shanghai, People’s Republic of China.

### Tissue dissociation and preparation

The fresh tumor tissue was stored in the sCelLiVE™ Tissue Preservation Solution (Singleron), and all the samples were transported to the Singleron laboratory at 2°C–8°C. The specimens were washed with Hank’s Balanced Salt Solution (HBSS) three times and minced into 1–2-mm pieces. Then, the tissue pieces were digested with 2 mL sCelLiVE™ Tissue Dissociation Solution (Singleron) at 37°C for 15 min in a 15-mL centrifuge tube with sustained agitation. After digestion, 40-μ sterile strainers were used to filter the samples, and the samples were centrifuged at 1,000 rpm for 5 min. Then, the supernatant was discarded, and the sediment was resuspended in 1 mL PBS (HyClone). The PBMCs were isolated by density gradient centrifugation using Ficoll-Paque Plus medium (GE Healthcare) and washed with Ca/Mg-free PBS. To remove the red blood cells, 2 mL GEXSCOPE™ red blood cell lysis buffer (Singleron) was added and incubated at 25°C for 10 min. The solution was then centrifuged at 500 × g for 5 min and suspended in PBS. The blood samples were centrifuged at 400 g for 5 min at 4°C, and the supernatant was discarded. After removing the red blood cells, PBMCs were isolated by centrifugation at 400 × g for 10 min at 4°C. The supernatant was discarded, and the PBMCs were resuspended in phosphate-buffered saline to obtain a single-cell suspension. The sample was stained with trypan blue (Sigma) and microscopically evaluated.

### Single-cell RNA sequencing

Single-cell suspensions at a concentration of 1×10^5^ cells/mL in PBS (HyClone) were prepared. They were then loaded onto microfluidic devices, and scRNA-seq libraries were constructed according to Singleron GEXSCOPE^®^ protocol by GEXSCOPE^®^ Single-Cell RNA Library Kit (Singleron Biotechnologies) (Dura et al., 2019). Individual libraries were diluted to 4 nM and pooled for sequencing. Pools were sequenced on Illumina HiSeq X with 150-bp paired end reads.

### Primary analysis of raw read data

Raw reads were processed using FastQC and fastp to remove low-quality reads. Poly-A tails and adaptor sequences were removed using Cutadapt. After quality control, the reads were mapped to the reference genome GRCh38 (Ensembl version 92 annotation) using STAR. Gene counts and UMI counts were acquired using featureCounts software (Liao et al., 2014). Expression matrix files for subsequent analyses were generated based on gene counts and UMI counts.

### Quality control, dimension reduction, and clustering

Cells were filtered by gene counts between 200 and 5,000 and UMI counts below 30,000. Cells with over 50% mitochondrial content were removed. After filtering, 80,680 cells were retained for the downstream analyses, with an average of 832 genes and 2,356 UMIs per cell. We used functions from Seurat v3.1.2 for dimension reduction and clustering (Satija et al., 2015). All gene expressions were normalized and scaled using NormalizeData and ScaleData. The top 2,000 variable genes were selected using FindVariableFeatures function for PCA. The cells were separated into 37 clusters using FindClusters, with the top 20 principal components and resolution parameter set at 1.2. For subclustering of epithelial cells, macrophages, M2 macrophages, and neutrophils, we set the resolution at 0.3, 0.5, 0.3, and 1.2, respectively. Uniform manifold approximation and projection (UMAP) algorithm was applied to visualize cells in a two-dimensional space. Harmony v1.0 was used to integrate samples and perform downstream analysis.

### Differentially expressed gene analysis

Genes expressed in more than 10% of the cells in a cluster and with an average log(Fold Change) greater than 0.25 were selected as differentially expressed genes (DEGs) using the FindMarkers function in Seurat v3.1.2 based on the Wilcox likelihood-ratio test with default parameters.

### Cell type annotation

The cell type identity of each cluster was determined with the expression of canonical markers found in the DEGs using the SynEcoSys^®^ database. Heatmaps displaying the expression of markers used to identify each cell type were generated using the DoHeatmap function in Seurat v3.1.2.

### Pathway enrichment analysis and pseudotime trajectory analysis

To investigate the potential functions of cellular subclusters, the Gene Ontology (GO) and Kyoto Encyclopedia of Genes and Genomes (KEGG) analyses were conducted using the “clusterProfiler” R package 3.16.1 (Yu et al., 2012). Pathways with a p_adj value less than 0.05 were considered significantly enriched. Gene Ontology gene sets including molecular function (MF), biological process (BP), and cellular component (CC) categories were used as references. Pseudotime trajectory analysis was performed using the Monocle2 package (V.2.18.0).

### scRNA-seq-based CNV detection

The InferCNV package was used to detect the CNVs in subclusters of epithelial malignant cells. Immune non-malignant cells were used as baselines to estimate the CNVs of malignant cells. Genes expressed in more than 20 cells were sorted based on their loci on each chromosome. The relative expression values were centered to 1, using a standard deviation of 1.5 from the residual-normalized expression values as the ceiling. A slide window size of 101 genes was used to smoothen the relative expression on each chromosome, to remove the effect of gene-specific expression.

### Cell–cell interaction analysis

CellCall v0.0.0.9000 was used to analyze the intercellular interaction based on the receptor–ligand interaction between two cellular clusters and infer the signaling pathways of the internal regulation (Zhang et al., 2021). The fraction of ligand–receptor gene interactions between cellular clusters was assessed by integrating the L2 norm of the receptor–ligand interaction and the activity fraction of downstream transcription factors (TFs), which was calculated by the inbuilt GSEA algorithm. Finally, ligand–receptor TFs with a significant interaction between cellular clusters were selected by the hypergeometric test and a *p*-value less than 0.05. Visualization was performed using the inbuilt plotting functions in CellCall.

### Immunohistochemistry staining (IHC)

Immunohistochemistry staining was performed on 4-μm-thick slices. Slides were incubated with primary antibodies including OMSR (1:100, Abmart), LRP6 (1:100, HuaAn Biotechnology), and SERPINF1 (1:50, Abmart), respectively. The slides were then incubated with the HRP-labeled secondary antibody and were visualized with diaminobenzidine. OSMR and LRP6 proteins were localized on the tumor cell membrane, and SERPINF1 was localized in the cytoplasm of stromal cells. The expression status was determined by the product score of the average percentage and intensity of positive cells under five random high-power fields. The score of percentage was as follows: <5% (0), 5%–25% (1), 25%–50% (2), 50%–75% (3), and >75% (4); for intensity: no staining (0), light brown (1), brown (2), and dark brown (3). OSMR (score of ≤ 3 and > 3), LRP6 (score of ≤ 6 and > 6), and SERPINF1 (score of ≤ 4 and > 4) were defined as low and high expression, respectively. The high expression of both SERPINF1 and LRP6 was identified as SERPINF1–LRP6 high, otherwise SERPINF1–LRP6 low.

### Statistical analysis

The correlation of the NLR with clinicopathological characteristics and treatment response of gastric cancer was analyzed with the one-way ANOVA test. The log-rank test in the Kaplan–Meier method and Cox proportional hazards model were used to analyze prognostic factors. The chi-squared test was used to analyze the categorical variables. A *p*-value < 0.05 was considered statistically significant. All tests were performed using SPSS 22.0 software (SPSS Inc.).

## Results

### Posttreatment NLR indicated poor outcomes of AGC patients treated with PD-1 antibody-based therapy

The clinical information of 45 stage IV AGC patients (33 male and 12 female patients) is listed in Table 1. PD-1 antibodies were administrated as second- or third-line therapy combined with anti-angiogenic agents for most patients. The median number of treatment cycles was five (range: 2 to 26). The median NLR at T0, T1, and T2 was 3.86, 2.83, and 3.45, respectively. No significant difference in the NLR at three timepoints was found among patients with different clinical characteristics including the number of metastatic organs, previous lines of treatment, and combination strategies.

**TABLE 1:** Clinicopathological characteristics of gastric cancer.

Clinicopathological characteristic		N	Percentage (%)
Gender	Male	33	73.3
	Female	12	26.7
Age	Median	67	
	Range	36–85	
Metastatic organs	1	27	60.0
	2	11	24.4
	≥3	7	15.6
Previous lines of treatment	0	3	6.7
	1	13	28.9
	2	29	64.4
Regimens			
ICI monotherapy		7	15.6
	Pembrolizumab	3	
	Camrelizumab	2	
	Toripalimab	2	
Chemotherapy plus ICIs		5	11.1
Metronomic capecitabine	Camrelizumab	5	
Targeted therapy plus ICIs		33	73.3
Apatinib	Camrelizumab	15	
	Nivolumab	2	
	Sintilimab	10	
Lenvatinib	Pembrolizumab	3	
Regorafenib	Nivolumab	2	
Trastuzumab	Sintilimab	1	
Cycles	Median	5	
	Range	2–26	

**FIGURE 1:**
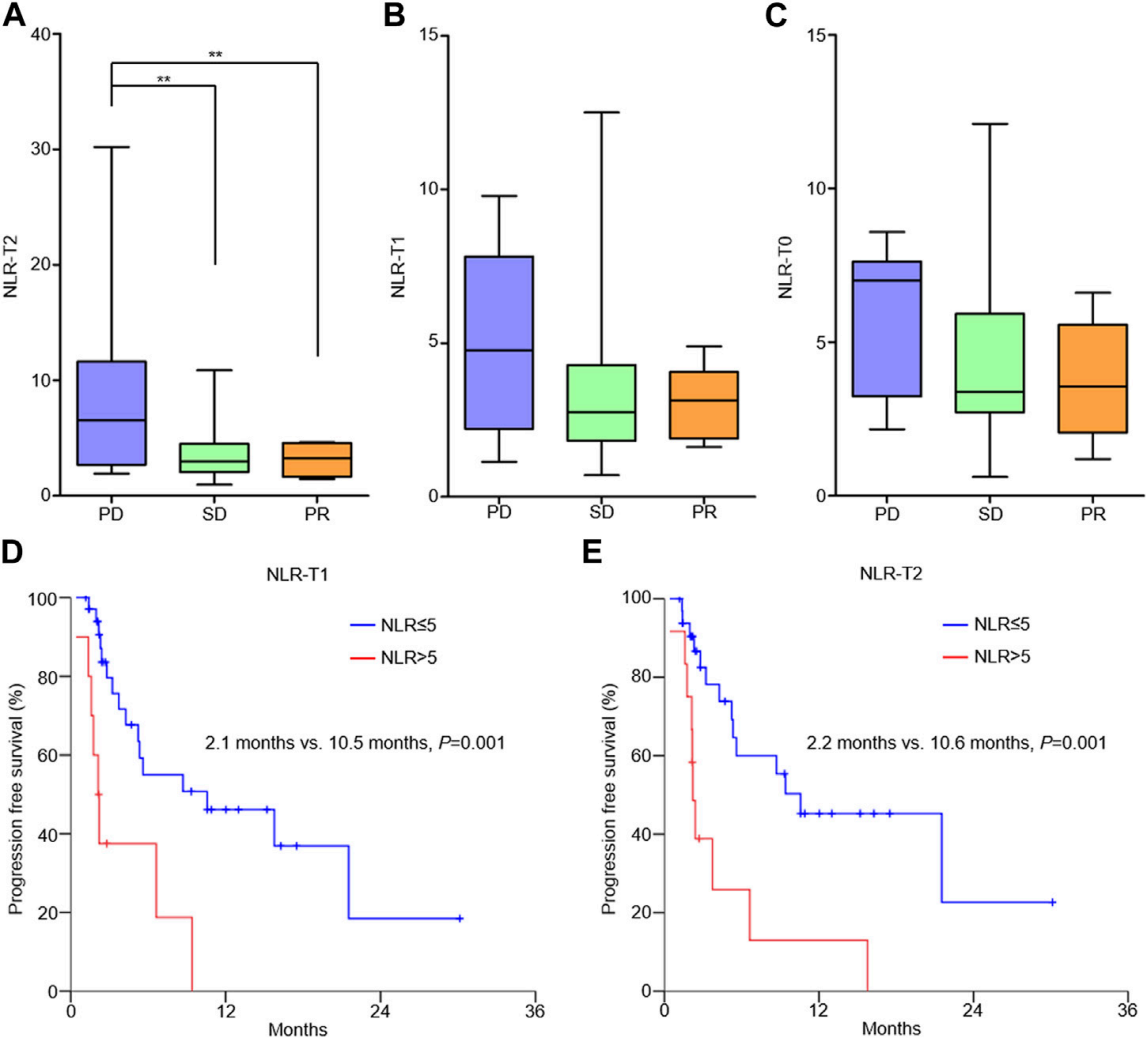
Correlation between NLR and outcomes of AGC patients receiving PD-1 antibody-based therapy. **(A)** NLR-T2 and patients’ objective response; **(B)** NLR-T1 and patients’ objective response; **(C)** NLR-T0 and patients’ objective response; **(D)** patients with high NLR-T1 had poorer PFS than those with low NLR-T1; and **(E)** patients with high NLR-T2 had poorer PFS than those with low NLR-T2.

NLR-T2 of patients with progressive disease (PD) was significantly higher than that of patients with stable disease (SD) and partial response (PR) (PD vs. SD, 8.74 ± 8.24 vs. 3.60 ± 2.40, *p* = 0.003; PD vs. PR, 8.74 ± 8.24 vs. 3.15 ± 1.47, *p* = 0.023; Figure 1A). The correlation between NLR-T1 and patient ORR and between NLR-T0 and patient ORR was not identified (Figures 1B, C). The median PFS of all patients was 2.8 months (0.4–30.1 months). A cutoff value of 5 of the NLR was selected according to the previous reference (Valero et al., 2021). Kaplan–Meier analysis showed that patients with high NLR-T1 had poorer PFS than those with low NLR-T1 (2.1 vs. 10.5 months, *p* = 0.001; Figure 1D), as well as for patients with high NLR-T2 (2.2 vs. 10.6 months, *p* = 0.001; Figure 1E). Multivariable analysis showed that high NLR-T2 was the independent prognostic factor of patient PFS (HR = 3.09, 95% CI 1.31–7.28, *p* = 0.010).

### Increased number of circulating neutrophils with high activity was detected in posttreatment peripheral blood samples

**FIGURE 2:**
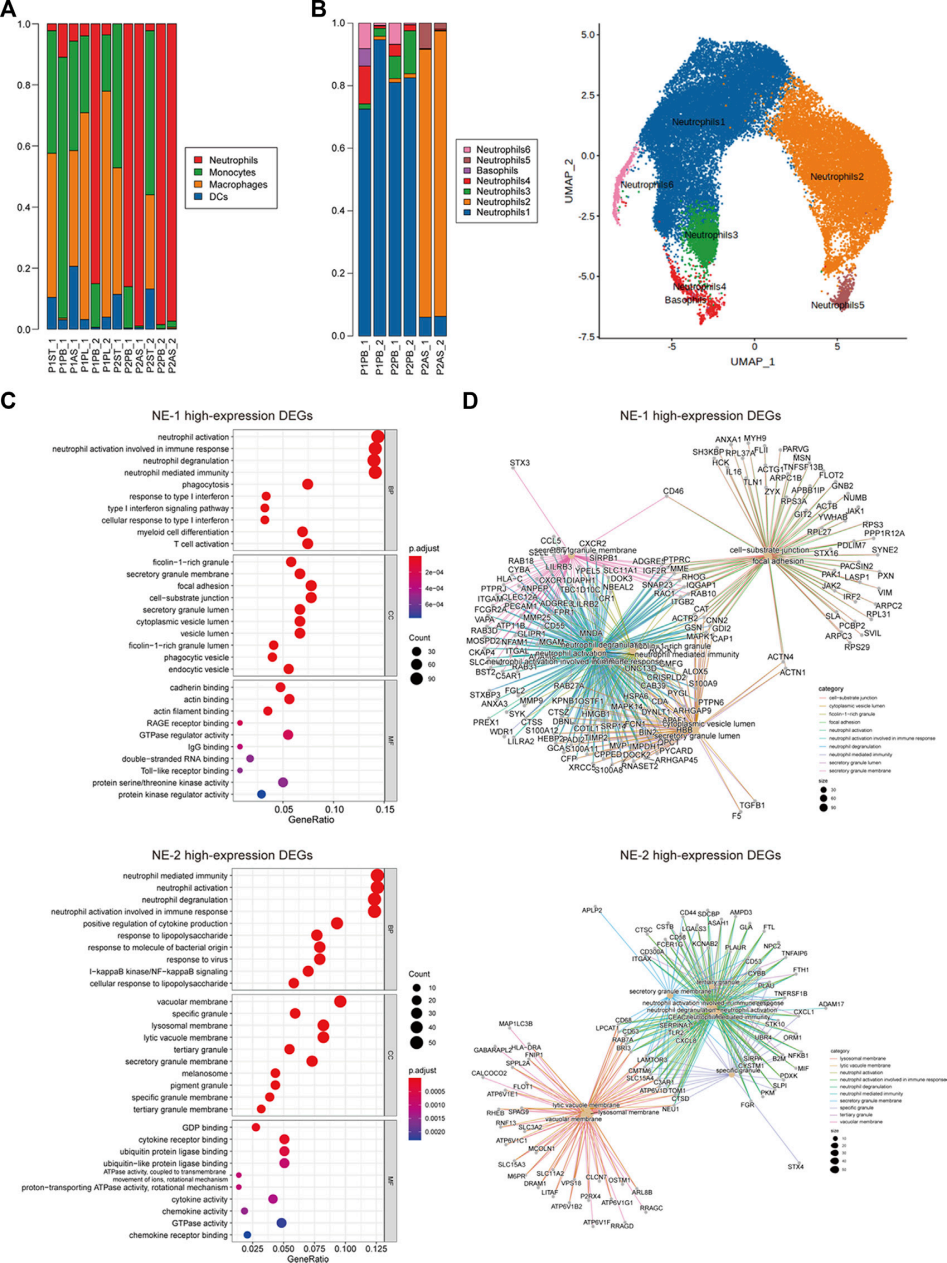
Cellular constitution of myeloid cells in peripheral blood samples and cellular subcluster analysis of neutrophils. **(A)** Cellular constitution of myeloid cells in peripheral blood samples; **(B)** cellular subcluster of neutrophils in peripheral blood and ascites samples; **(C)** dot plots of GO analysis of high-expression DEGs of neutrophil cluster 1 (NE-1) and cluster 2 (NE-2); and **(D)** net plots of GO analysis of high-expression DEGs of NE-1 and NE-2.

A total of 80,680 cells from 12 samples were identified. The number of cells in each sample is given in Supplementary Table S2. Endothelial cells, epithelial cells, myeloid cells, myofibroblasts, pleural mesothelial cells, pericytes, plasma cells, platelets, T cells, plasmacytoid dendritic cells, and B cells were annotated by established marker genes (Supplementary Figures S1B, C). Myeloid cells of all samples were then divided into neutrophils, monocytes, macrophages, and dendritic cells (Supplementary Table S3). Neutrophils were mainly detected in peripheral blood (PB) samples of both patients and ascites (AS) samples of P2, while tumor-infiltrating neutrophils were barely detected in stomach tumor (ST) samples of two patients. The proportion and number of neutrophils were both increased in posttreatment PB samples (Figure 2A; Supplementary Table S4). Neutrophils were further divided into seven cellular subclusters. Neutrophil cluster 1 (NE-1) was the major subcluster in PB samples, and NE-2 was mainly detected in AS samples of P2 (Figure 2B).

Biological functions of high-expression DEGs of NE-1 and NE-2 were analyzed by GO enrichment analysis. Neutrophil activation, neutrophil activation involved in immune response, neutrophil degranulation, and neutrophil-mediated immunity were the top enriched biological processes (BPs) of both NE-1 and NE-2 (Figure 2C). For molecular functions (MFs), cadherin binding, actin binding, and actin filament binding were the top three MFs enriched in NE-1, while GDP binding, cytokine–receptor binding, and ubiquitin protein ligase binding were the top three MFs enriched in NE-2 (Figure 2C). Cancer-promoting genes including *MMP9*, *S100A8*, *S100A9*, *PORK2*, and *TGF-β1* were identified as high-expression DEGs in NE-1 (Figure 2D; Supplementary Table S5).

### Circulating neutrophil subcluster NE-1 displayed an intermediate differentiation state with high chemotaxis ability

**FIGURE 3:**
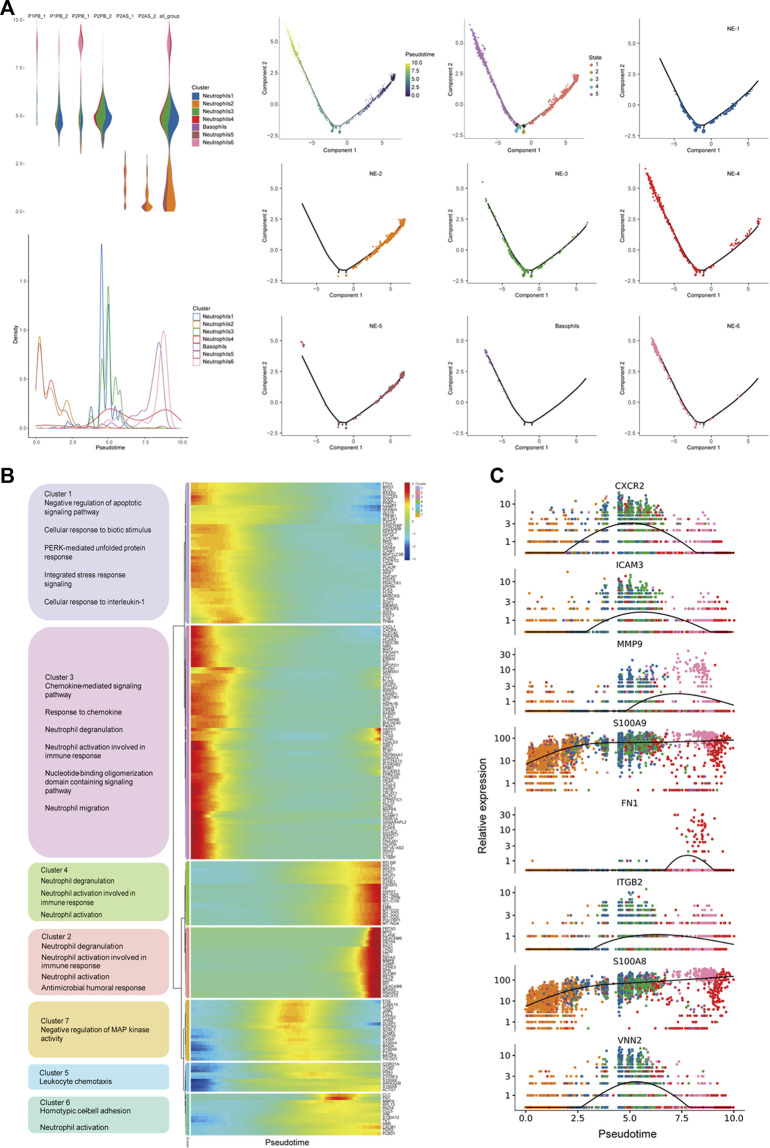
Pseudotime trajectory analysis revealed molecular states of circulating neutrophils. **(A)** Constitution of the pseudotime trajectory of neutrophils in peripheral blood and ascites samples; **(B)** cluster analysis and function enrichment of genes with significant expression changes; and **(C)** dynamic expression of top changed genes of neutrophils.

To further explore the features of neutrophil subclusters, the pseudotime trajectory of neutrophils was constituted. NE-1 and NE-3 displayed intermediate states, while NE-2/NE-5 and NE-4/NE-6 were at two ends of the trajectory, respectively (Figure 3A). Genes with significant expression changes were clustered into seven groups. Genes of clusters 5, 6, and 7 were enriched at the intermediate stage. Pathway enrichment analysis revealed that genes of cluster 5 were associated with leukocyte chemotaxis, genes of cluster 6 were associated with homotypic cell–cell adhesion, and genes of cluster 7 were associated with the negative regulation of MAP kinase activity (Figure 3B). Dynamic expression of the top changed genes in these three clusters, including *CXCR2*, *ICAM3*, *MMP9*, *S100A9*, *FN1*, *ITGB2*, *S100A8,* and *VNN2*, could be identified (Figure 3C).

### Subclusters of malignant epithelial cells and M2 macrophages participated in neutrophil activation

**FIGURE 4:**
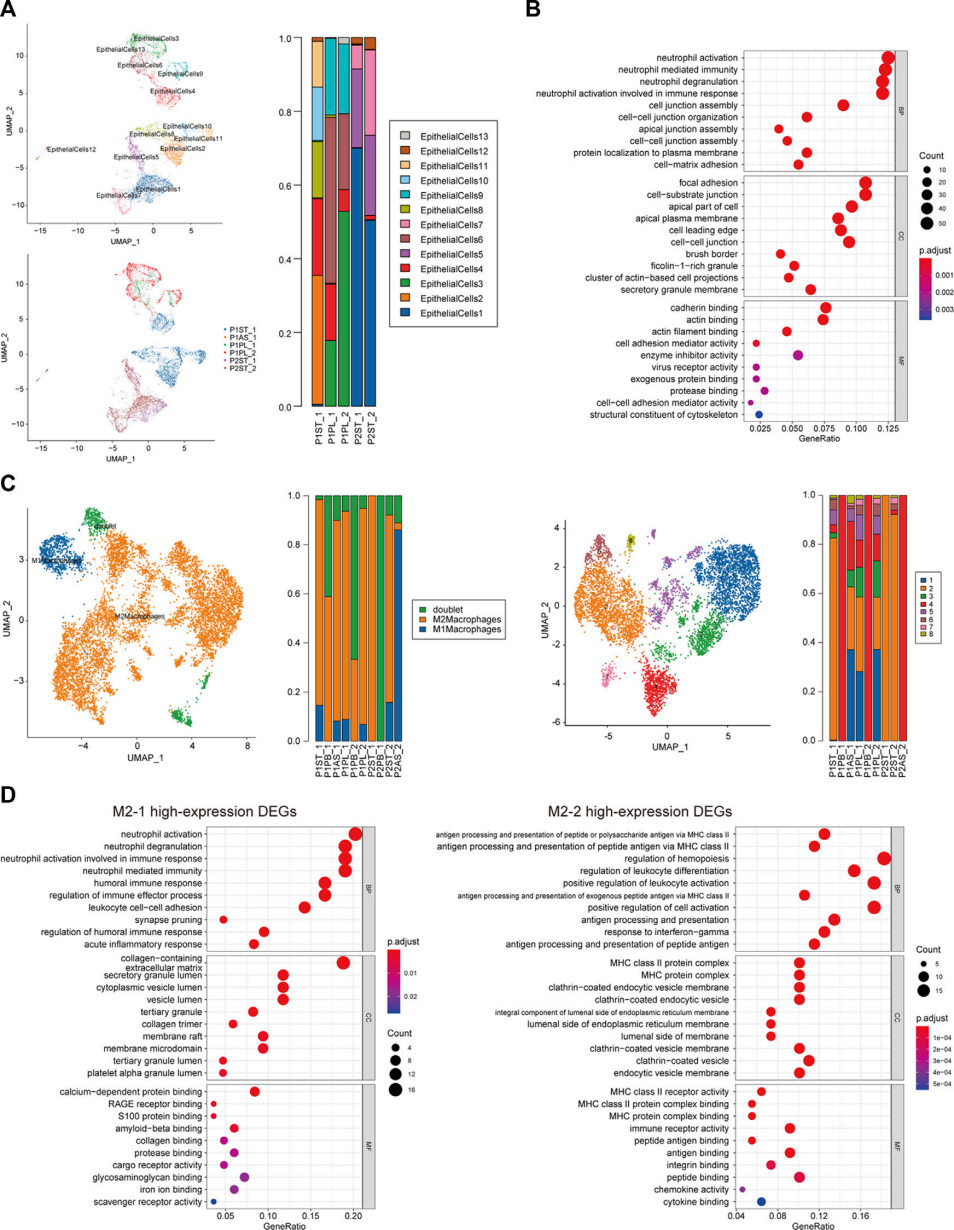
Cellular subcluster analysis of malignant epithelial cells and M2 macrophages. **(A)** Cellular subcluster of malignant epithelial cells in primary lesion and pleural fluid samples; **(B)** dot plot of GO analysis of high-expression DEGs of malignant epithelial cell cluster 4 (EP-4); **(C)** cellular constitution of macrophages and subclusters of M2 macrophages; and **(D)** dot plots of GO analysis of high-expression DEGs of M2 macrophage cluster 1 (M2-1) and cluster 2 (M2-2).

Malignant epithelial cells (EPs) were identified by inferred CNV algorithm and were divided into 13 cellular subclusters (Supplementary Figure S2; Supplementary Table S6). The distribution of subclusters in P1 and P2 was different, while subcluster 4 of malignant epithelial cells (EP-4) was found in both patients, especially as a new subcluster of P2 after treatment (Figure 4A). The DEGs of EP-4 are listed in Supplementary Table S7. The top three enriched BPs of high-expression DEGs of EP-4 were neutrophil activation, neutrophil-mediated immunity, and neutrophil degranulation. The top three enriched MFs of EP-4 were cadherin binding, actin binding, and actin filament binding (Figure 4B).

In all non-peripheral blood samples, macrophages accounted for a major part (Figure 2A). The proportion of M2 macrophages in most samples was significantly higher than that of M1 macrophages (Figure 4C). M2 macrophages could be subdivided into eight cellular subclusters (Supplementary Table S8). M2 macrophage cluster 1 (M2-1) was only found in samples of P1, while M2-2 was found in samples of both patients (Figure 4C). The DEGs of M2-1 and M2-2 are listed in Supplementary Table S9 and Supplementary Table S10, respectively. The top three enriched BPs of high-expression DEGs of M2-1 were neutrophil activation, neutrophil degranulation, and neutrophil activation involved in immune response. Meanwhile, the high-expression DEGs of M2-2 were enriched in the regulation of leukocyte differentiation and positive regulation of leukocyte activation (Figure 4D).

### Crosstalk among circulating neutrophils, M2 macrophages, and malignant epithelial cells attributed to tumor progression

**FIGURE 5:**
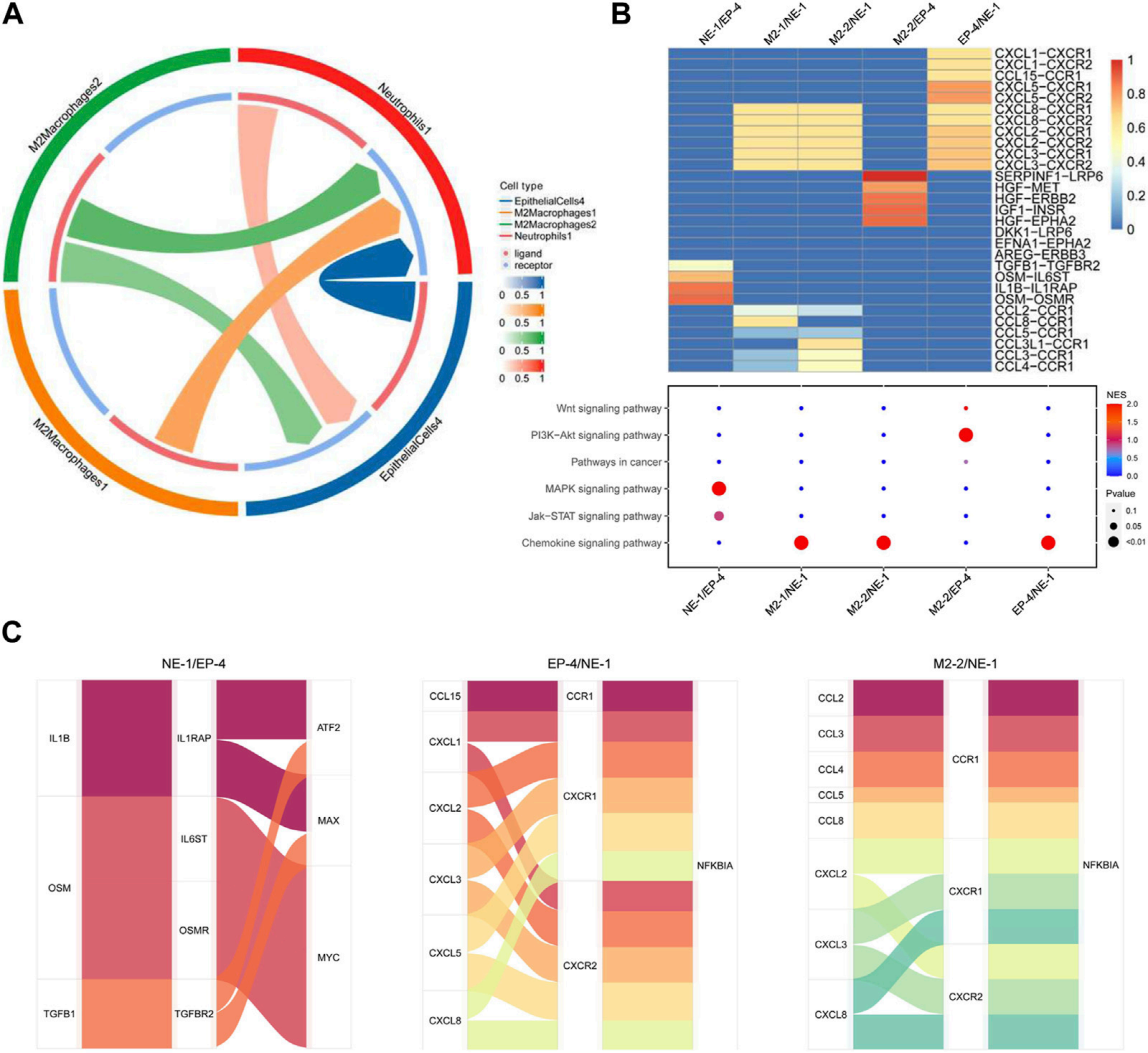
Cellular interaction analysis among neutrophils, malignant epithelial cells, and M2 macrophages. **(A)** Cell network of NE-1, EP-4, M2-1, and M2-2 was analyzed using CellCall; **(B)** heatmap of ligand–receptor interactions among NE-1, EP-4, M2-1, and M2-2. Signaling pathways involved in ligand–receptor interactions were analyzed KEGG enrichment analysis; **(C)** Sankey diagram displayed transcript factors downstream of the signaling pathway.

Cellular interactions among neutrophils, M2 macrophages, and malignant epithelial cells were analyzed (Supplementary Figure S3). The results of receptor–ligand interaction analysis showed that receptors of NE-1 could correspond to ligands from EP-4, M2-1, and M2-2. Ligands from NE-1 also interacted with receptors of EP-4 (Figure 5A). M2-1, M2-2, and EP-4 interacted with NE-1 via CXCL8-CXCR1/2, CXCL2-CXCR1/2, and CXCL3-CXCR1/2 axes, respectively. Meanwhile, NE-1 could interact with EP-4 via OSM-OSMR, IL1B-IL1RAP, OSM-IL6ST, and TGFB1-TGFBR2 axes. KEGG enrichment analysis showed that ligand–receptor interactions between NE-1 and EP-4 were enriched in the MAPK signaling pathway and Jak-STAT signaling pathway (Figure 5B). M2-2 could interact with EP-4 via SERPINF1-LRP6, HGF-MET, HGF-ERBB2, IGF1-INSR, and HGF-EPHA2 axes. Using the Sankey diagram, transcript factors ATF2, MAX, and MYC were found to participate in NE-1/EP-4 regulation. NFKBIA was the transcript factor that participated in EP-4/NE-1 and M2-2/NE-1 regulation (Figure 5C).

**FIGURE 6:**
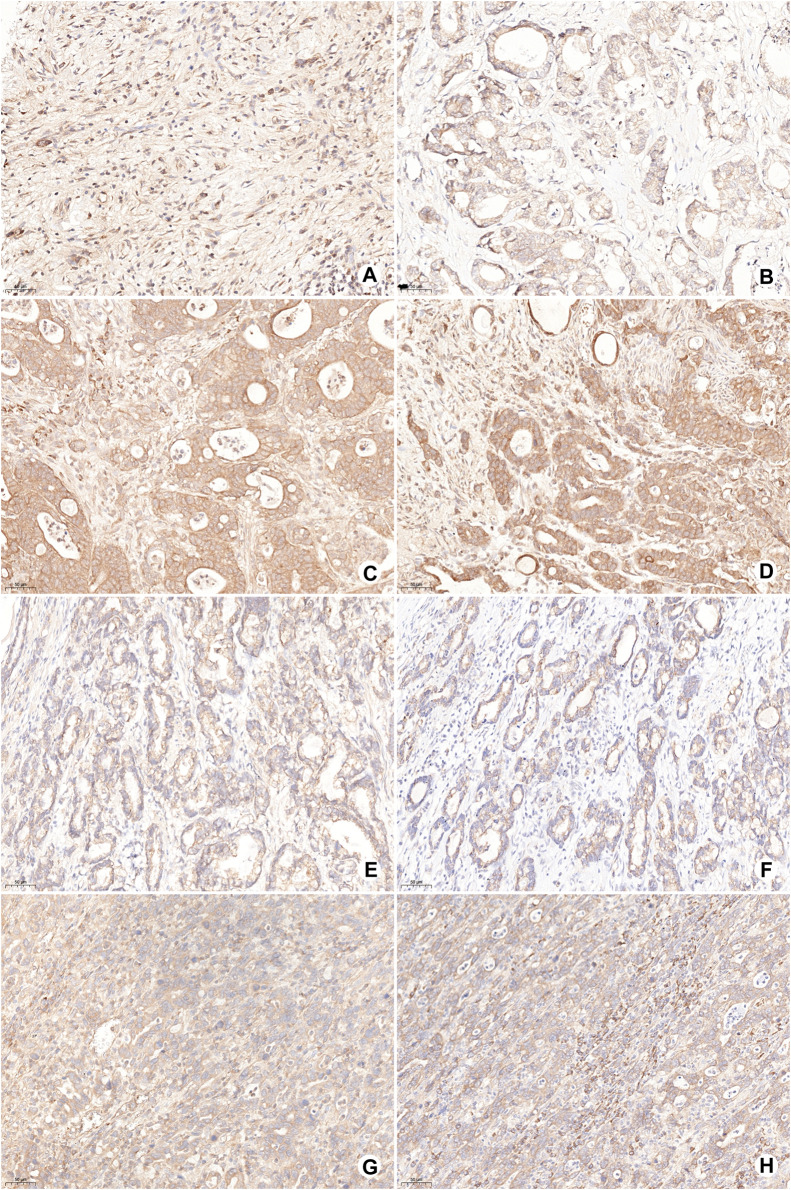
Representative images of OSMR, SERPINF1, and LRP6 expression in gastric cancer samples by IHC. **(A,B)** Low expression of OSMR in tumor cells; **(C,D)** high expression of OSMR in tumor cells; **(E)** low expression of LRP6 in tumor cells; **(F)** low expression of SERPINF1 in stromal cells; **(G)** high expression of LRP6 in tumor cells; and **(H)** high expression of SERPINF1 in stromal cells.

The expression of OSMR and LRP6 in tumor cells and SERPINF1 in stromal cells was detected by IHC. OSMR, LRP6, and SERPINF1 were expressed in most gastric cancer samples. The low expression of OSMR was detected in 15 cases (17%; Figures 6A, B) and high expression in 73 cases (83%; Figures 6C, D). The high expression of OSMR was significantly correlated with the N stage (*p* = 0.010; Table 2). A total of 53 samples with either or both SERPINF1- and LRP6-low expression were identified as SERPINF1–LRP6-low expression (Figures 6E, F), while SERPINF1–LRP6-high expression was detected in 35 samples (Figures 6G, H). SERPINF1–LRP6-high expression was also significantly correlated with the N stage (*p* = 0.018; Table 2).

**TABLE 2:** Correlations of OSMR and SERPINF1–LRP6 with clinicopathological characteristics of gastric cancer.

Clinicopathological characteristic		OSMR		P	SERPINF1–LRP6		P
		Low (%)	High (%)		Low (%)	High (%)	
Tumor positions	Cardia	2 (12.5)	14 (87.5)	0.700	6 (37.5)	10 (62.5)	0.094
	Corpus	6 (15.4)	33 (84.6)		27 (69.2)	12 (30.8)	
	Antrum	7 (21.2)	26 (78.8)		20 (60.6)	13 (39.4)	
Borrmann typing	I	0 (0)	6 (100.0)	0.290	5 (83.3)	1 (16.7)	0.619
	II	3 (14.3)	18 (85.7)		13 (61.9)	8 (38.1)	
	III	11 (22.0)	39 (78.0)		29 (58.0)	21 (42.0)	
	IV	1 (9.1)	10 (83.0)		6 (54.5)	5 (45.5)	
T stage	T1–2	2 (13.3)	13 (86.7)	0.966	11 (73.3)	4 (26.7)	0.255
	T3–4	13 (17.8)	60 (82.2)		42 (57.5)	31 (42.5)	
N stage	N0	7 (38.9)	11 (61.1)	0.010	16 (88.9)	2 (11.1)	0.018
	N1–2	6 (17.6)	28 (82.4)		19 (55.9)	15 (44.1)	
	N3	2 (5.6)	34 (94.4)		18 (50.0)	18 (50.0)	
TNM staging	I	1 (14.3)	6 (85.7)	0.076	6 (85.7)	1 (14.3)	0.069
	II	8 (32.0)	17 (68.0)		18 (72.0)	7 (28.0)	
	III	6 (10.7)	50 (89.3)		29 (51.8)	27 (48.2)	

## Discussion

In the present study, the correlation between an elevated NLR and clinical outcome of AGC patients treated with PD-1 antibody-based therapy was reviewed and analyzed. Then, scRNA-seq analysis was performed based on pre- and posttreatment samples to explore the underlying role of elevated circulating neutrophils during tumor progression. The results showed that an elevated subcluster of circulating neutrophils expressed high-activity phenotypes including neutrophil activation and chemotaxis. High expression of pro-tumor genes was detected in this subcluster. Close interactions among subclusters of circulating neutrophils, M2 macrophages, and malignant epithelial cells were found and could participate in gastric cancer progression during ICI treatment.

NLR as a prognostic biomarker has been identified in multiple kinds of tumors. In a study on gastric cancer, Sun et al. (2016) reviewed 19 studies including 5,421 patients with different stages who were treated with chemotherapy and/or surgical resection and showed that an elevated pretreatment NLR was a negative prognostic biomarker for patients’ outcomes. For ICI treatment, the value of NLR as a prognostic biomarker in gastric cancer is under investigation. A total of 71 gastric cancer patients were enrolled in a pan-cancer investigation which assessed the correlation between pretreatment NLR and efficacy of ICI treatment. The confidence interval of the hazard ratio of pretreatment NLR crossed 1.0 for the gastric cancer subgroup (Valero et al., 2021). A retrospective study showed that NLR was an independent prognosis biomarker of AGC patients who received anti-PD-1 treatment (Gou et al., 2021). In the present study, we found that the posttreatment NLR was significantly correlated with ORR and PFS of PD-1 antibody-based therapeutics of AGC patients.

Currently, both pre- and posttreatment NLRs were reported to be associated with patient prognosis in different studies. For metastatic renal cell carcinoma, NLR at 6 weeks after treatment was indicated as a stronger predictor than NLR at baseline (Lalani et al., 2018). Pretreatment NLR was associated with the outcome of lung cancer and melanoma treated using ICIs (Capone et al., 2018; Fukui et al., 2019). All these studies including the present study were retrospective investigations. The heterogeneous phenomenon about pre- and posttreatment NLR may contribute to the different sample sizes, tumor types, and therapeutics. Despite heterogeneity, combined with our results, NLR could be a convenient method to indicate early the outcome of AGC patients treated with PD-1 antibody-based therapy.

The impact of TANs infiltrating into the tumor microenvironment on tumor initiation and progression has been recognized in the past few years, which can promote malignant behaviors of tumor cells by producing reactive oxygen species, cytokine, proteinase, and angiogenesis factors (Coffelt et al., 2016; Mollinedo, 2019). However, the role of circulating neutrophils during the progression of ICI treatment of gastric cancer was barely investigated. In the present study, a subcluster of neutrophils with high-activity phenotypes accounted for a major proportion of PB samples after treatment, which also highly expressed well-known tumor-promoting genes of gastric cancer including *MMP9*, *S100A8*, *S100A9*, *PORK2*, and *TGF-β1* (Verma et al., 2015; Zhang et al., 2018; Sprenkeler et al., 2022). This subcluster of circulating neutrophils (NE-1) also possessed a high chemotaxis revealed by pseudotime trajectory analysis. These results indicated that elevated circulating neutrophils could promote tumor progression via the endocrine pathway by releasing pro-tumor factors and could be the major source of TANs.

The interaction between tumor cells and host immune microenvironment plays an important role in tumor progression and treatment resistance. Tumor cells can also participate in recruiting and activating neutrophils (Mantovani et al., 2011; Coffelt et al., 2016). EP-4, a common subcluster of malignant epithelial cells in both patients, displayed a high neutrophil-activating phenotype, which might attribute to the activation and infiltration of circulating neutrophils. PD-1 antibodies mainly target immune cells infiltrating into the tumor microenvironment (Wei et al., 2017). Tumor-infiltrating CD8^+^ T cells, which are the major therapeutic target of PD-1 antibodies, are not found in non-peripheral blood samples of the two patients in this study (Supplementary Figure S1B). On the other hand, dendritic cells and macrophages, which are reported to interact with PD-1 antibodies, can be found in non-peripheral blood samples (Shergold et al., 2019; Mayoux et al., 2020). M2 macrophage infiltration is a negative factor that can impair treatment efficacy of PD-1 antibodies (Camelliti et al., 2020). In the present study, M2 macrophages accounted for a significant proportion of tumor-infiltrated immune cells, and two major subclusters of M2 macrophages possessed a neutrophil-activating phenotype. Therefore, both tumor cells and M2 macrophages could promote the activation and infiltration of circulating neutrophils into the tumor microenvironment during treatment.

Cell–cell interaction analysis further revealed the molecular pathways of interactions among circulating neutrophils, tumor cells, and M2 macrophages. Subclusters of tumor cells and M2 macrophages regulate NE-1 via chemokine signaling pathways including CXCL8-CXCR1/2, CXCL2-CXCR1/2, and CXCL3-CXCR1/2 axes. These axes not only lead to neutrophil recruitment but also participate in tumor promotion by activating neutrophils to increase the expression of pro-tumor factors like MMP-9 from neutrophils (Bonecchi et al., 2022). OSM and IL1B provided by NE-1 could regulate the Jak-STAT signaling pathway and MAPK signaling pathway of EP-4, respectively. OSM was found to participate in regulating malignant behaviors of multiple tumors including gastric cancer (Yu et al., 2019). IHC analysis showed that the high expression of OMSR was detected in gastric cancer samples and was correlated with lymph node metastasis. The activation of the Jak-STAT pathway in tumor cells after PD-1 antibody treatment could stimulate MDM2 expression and induce treatment resistance (Arasanz et al., 2021). In addition to neutrophils, subclusters of M2 macrophages also participated in regulating the activation of tumor cells. Co-expression of stromal SERPINF1 and tumor cell LRP6, which is one of the ligand–receptor interactions of M2 macrophages and tumor cells, was correlated with lymph node metastasis in gastric cancer samples. High expression of SERPINF1 was detected in gastric cancer patients with poor prognosis and was correlated with immune cell infiltration (Lee et al., 2022; Wang et al., 2022). The interaction between SERPINF1 and LRP6 has not been reported in gastric cancer. LRP6 is a receptor interacting with the Wnt/β-catenin signaling pathway and participated in regulating cell proliferation and migration of tumor cells (Alrefaei and Abu-Elmagd, 2022). The expression of LRP6 had no correlation with the clinical characteristics of gastric cancer patients, while its co-expression with SERPINF1 was correlated with the cancer malignant phenotype, which indicated the role of SERPINF1–LRP6 interaction during tumor progression. Therefore, by using bioinformatics analysis and IHC detection, these results indicated that interactions among circulating neutrophils, tumor cells, and M2 macrophages can promote disease progression of gastric cancer and may attribute to resistance of PD-1 antibody-based treatment.

## Conclusion

Posttreatment NLR could be an early prognostic biomarker of AGC patients receiving PD-1 antibody-based therapy. Circulating neutrophils could be activated by both tumor cells and M2 macrophages; in turn, circulating neutrophils and M2 macrophages could regulate critical tumor-promoting pathways, which could attribute to tumor progression after PD-1 antibody treatment of AGC patients.

## Data Availability

The original contributions presented in the study are publicly available. This data can be found here (http://www.ncbi.nlm.nih.gov/bioproject/975683). The BioProject ID is PRJNA975683.

## References

[B1] Alrefaei A. F. Abu-Elmagd M. (2022). LRP6 receptor plays essential functions in development and human diseases. Genes (Basel). 13 (1), 120. 10.3390/genes13010120 35052459 PMC8775365

[B2] Arasanz H. Zuazo M. Bocanegra A. Chocarro L. Blanco E. Martinez M. (2021). Hyperprogressive disease: Main features and key controversies. Int. J. Mol. Sci. 22 (7), 3736. 10.3390/ijms22073736 33916696 PMC8038385

[B3] Bai R. Lv Z. Xu D. Cui J. (2020). Predictive biomarkers for cancer immunotherapy with immune checkpoint inhibitors. Biomark. Res. 8, 34. 10.1186/s40364-020-00209-0 32864131 PMC7450548

[B4] Bonecchi R. Mantovani A. Jaillon S. (2022). Chemokines as regulators of neutrophils: Focus on tumors, therapeutic targeting, and immunotherapy. Cancers (Basel) 14 (3), 680. 10.3390/cancers14030680 35158948 PMC8833344

[B5] Camelliti S. Le Noci V. Bianchi F. Moscheni C. Arnaboldi F. Gagliano N. (2020). Mechanisms of hyperprogressive disease after immune checkpoint inhibitor therapy: What we (don't) know. J. Exp. Clin. Cancer Res. 39 (1), 236. 10.1186/s13046-020-01721-9 33168050 PMC7650183

[B6] Capone M. Giannarelli D. Mallardo D. Madonna G. Festino L. Grimaldi A. M. (2018). Baseline neutrophil-to-lymphocyte ratio (NLR) and derived NLR could predict overall survival in patients with advanced melanoma treated with nivolumab. J. Immunother. Cancer 6 (1), 74. 10.1186/s40425-018-0383-1 30012216 PMC6048712

[B7] Coffelt S. B. Wellenstein M. D. de Visser K. E. (2016). Neutrophils in cancer: Neutral no more. Nat. Rev. Cancer 16 (7), 431–446. 10.1038/nrc.2016.52 27282249

[B8] Cunningham D. Starling N. Rao S. Iveson T. Nicolson M. Coxon F. (2008). Capecitabine and oxaliplatin for advanced esophagogastric cancer. N. Engl. J. Med. 358 (1), 36–46. 10.1056/NEJMoa073149 18172173

[B9] Dura B. Choi J. Y. Zhang K. Damsky W. Thakral D. Bosenberg M. (2019). scFTD-seq: freeze-thaw lysis based, portable approach toward highly distributed single-cell 3' mRNA profiling. Nucleic Acids Res. 47 (3), e16. 10.1093/nar/gky1173 30462277 PMC6379653

[B10] Fukui T. Okuma Y. Nakahara Y. Otani S. Igawa S. Katagiri M. (2019). Activity of nivolumab and utility of neutrophil-to-lymphocyte ratio as a predictive biomarker for advanced non-small-cell lung cancer: A prospective observational study. Clin. Lung Cancer 20 (3), 208–214. 10.1016/j.cllc.2018.04.021 29803573

[B11] Fukuoka S. Hara H. Takahashi N. Kojima T. Kawazoe A. Asayama M. (2020). Regorafenib plus nivolumab in patients with advanced gastric or colorectal cancer: An open-label, dose-escalation, and dose-expansion phase ib trial (REGONIVO, EPOC1603). J. Clin. Oncol. 38 (18), 2053–2061. 10.1200/JCO.19.03296 32343640

[B12] Gao K. Wu J. (2019). National trend of gastric cancer mortality in China (2003-2015): A population-based study. Cancer Commun. (Lond) 39 (1), 24. 10.1186/s40880-019-0372-x 31046840 PMC6498569

[B13] Gou M. Qu T. Wang Z. Yan H. Si Y. Zhang Y. (2021). Neutrophil-to-Lymphocyte ratio (NLR) predicts PD-1 inhibitor survival in patients with metastatic gastric cancer. J. Immunol. Res. 2021, 2549295. 10.1155/2021/2549295 34993252 PMC8727102

[B14] Hanahan D. (2022). Hallmarks of cancer: New dimensions. Cancer Discov. 12 (1), 31–46. 10.1158/2159-8290.CD-21-1059 35022204

[B15] Jaillon S. Ponzetta A. Di Mitri D. Santoni A. Bonecchi R. Mantovani A. (2020). Neutrophil diversity and plasticity in tumour progression and therapy. Nat. Rev. Cancer 20 (9), 485–503. 10.1038/s41568-020-0281-y 32694624

[B16] Janjigian Y. Y. Kawazoe A. Yanez P. Li N. Lonardi S. Kolesnik O. (2021). The KEYNOTE-811 trial of dual PD-1 and HER2 blockade in HER2-positive gastric cancer. Nature 600 (7890), 727–730. 10.1038/s41586-021-04161-3 34912120 PMC8959470

[B17] Janjigian Y. Y. Shitara K. Moehler M. Garrido M. Salman P. Shen L. (2021). First-line nivolumab plus chemotherapy versus chemotherapy alone for advanced gastric, gastro-oesophageal junction, and oesophageal adenocarcinoma (CheckMate 649): A randomised, open-label, phase 3 trial. Lancet 398 (10294), 27–40. 10.1016/S0140-6736(21)00797-2 34102137 PMC8436782

[B18] Kang Y. K. Boku N. Satoh T. Ryu M. H. Chao Y. Kato K. (2017). Nivolumab in patients with advanced gastric or gastro-oesophageal junction cancer refractory to, or intolerant of, at least two previous chemotherapy regimens (ONO-4538-12, ATTRACTION-2): A randomised, double-blind, placebo-controlled, phase 3 trial. Lancet 390 (10111), 2461–2471. 10.1016/S0140-6736(17)31827-5 28993052

[B19] Kundel Y. Sternschuss M. Moore A. Perl G. Brenner B. Goldvaser H. (2020). Efficacy of immune-checkpoint inhibitors in metastatic gastric or gastroesophageal junction adenocarcinoma by patient subgroups: A systematic review and meta-analysis. Cancer Med. 9 (20), 7613–7625. 10.1002/cam4.3417 32869544 PMC7571828

[B20] Lalani A. A. Xie W. Martini D. J. Steinharter J. A. Norton C. K. Krajewski K. M. (2018). Change in Neutrophil-to-lymphocyte ratio (NLR) in response to immune checkpoint blockade for metastatic renal cell carcinoma. J. Immunother. Cancer 6 (1), 5. 10.1186/s40425-018-0315-0 29353553 PMC5776777

[B21] Lee M. Cho H. J. Park K. S. Jung H. Y. (2022). ELK3 controls gastric cancer cell migration and invasion by regulating ECM remodeling-related genes. Int. J. Mol. Sci. 23 (7), 3709. 10.3390/ijms23073709 35409069 PMC8998440

[B22] Liao Y. Smyth G. K. Shi W. (2014). featureCounts: an efficient general purpose program for assigning sequence reads to genomic features. Bioinformatics 30 (7), 923–930. 10.1093/bioinformatics/btt656 24227677

[B23] Mantovani A. Cassatella M. A. Costantini C. Jaillon S. (2011). Neutrophils in the activation and regulation of innate and adaptive immunity. Nat. Rev. Immunol. 11 (8), 519–531. 10.1038/nri3024 21785456

[B24] Mayoux M. Roller A. Pulko V. Sammicheli S. Chen S. Sum E. (2020). Dendritic cells dictate responses to PD-L1 blockade cancer immunotherapy. Sci. Transl. Med. 12 (534), eaav7431. 10.1126/scitranslmed.aav7431 32161104

[B25] Mollinedo F. (2019). Neutrophil degranulation, plasticity, and cancer metastasis. Trends Immunol. 40 (3), 228–242. 10.1016/j.it.2019.01.006 30777721

[B26] Ocana A. Nieto-Jimenez C. Pandiella A. Templeton A. J. (2017). Neutrophils in cancer: Prognostic role and therapeutic strategies. Mol. Cancer 16 (1), 137. 10.1186/s12943-017-0707-7 28810877 PMC5558711

[B27] Satija R. Farrell J. A. Gennert D. Schier A. F. Regev A. (2015). Spatial reconstruction of single-cell gene expression data. Nat. Biotechnol. 33 (5), 495–502. 10.1038/nbt.3192 25867923 PMC4430369

[B28] Sharma P. Siddiqui B. A. Anandhan S. Yadav S. S. Subudhi S. K. Gao J. (2021). The next decade of immune checkpoint therapy. Cancer Discov. 11 (4), 838–857. 10.1158/2159-8290.CD-20-1680 33811120

[B29] Shergold A. L. Millar R. Nibbs R. J. B. (2019). Understanding and overcoming the resistance of cancer to PD-1/PD-L1 blockade. Pharmacol. Res. 145, 104258. 10.1016/j.phrs.2019.104258 31063806

[B30] Sprenkeler E. G. G. Zandstra J. van Kleef N. D. Goetschalckx I. Verstegen B. Aarts C. E. M. (2022). S100A8/A9 is a marker for the release of neutrophil extracellular traps and induces neutrophil activation. Cells 11 (2), 236. 10.3390/cells11020236 35053354 PMC8773660

[B31] Sun J. Chen X. Gao P. Song Y. Huang X. Yang Y. (2016). Can the neutrophil to lymphocyte ratio Be used to determine gastric cancer treatment outcomes? A systematic review and meta-analysis. Dis. Markers 2016, 7862469. 10.1155/2016/7862469 26924872 PMC4746375

[B32] Valero C. Lee M. Hoen D. Weiss K. Kelly D. W. Adusumilli P. S. (2021). Pretreatment neutrophil-to-lymphocyte ratio and mutational burden as biomarkers of tumor response to immune checkpoint inhibitors. Nat. Commun. 12 (1), 729. 10.1038/s41467-021-20935-9 33526794 PMC7851155

[B33] Verma S. Kesh K. Gupta A. Swarnakar S. (2015). An overview of matrix metalloproteinase 9 polymorphism and gastric cancer risk. Asian Pac J. Cancer Prev. 16 (17), 7393–7400. 10.7314/apjcp.2015.16.17.7393 26625734

[B34] Wang F. H. Shen L. Li J. Zhou Z. W. Liang H. Zhang X. T. (2019). The Chinese society of clinical Oncology (CSCO): Clinical guidelines for the diagnosis and treatment of gastric cancer. Cancer Commun. (Lond) 39 (1), 10. 10.1186/s40880-019-0349-9 30885279 PMC6423835

[B35] Wang J. Xia W. Huang Y. Li H. Tang Y. Li Y. (2022). A vasculogenic mimicry prognostic signature associated with immune signature in human gastric cancer. Front. Immunol. 13, 1016612. 10.3389/fimmu.2022.1016612 36505458 PMC9727221

[B36] Wei S. C. Levine J. H. Cogdill A. P. Zhao Y. Anang N. A. S. Andrews M. C. (2017). Distinct cellular mechanisms underlie anti-CTLA-4 and anti-PD-1 checkpoint blockade. Cell 170 (6), 1120–1133. 10.1016/j.cell.2017.07.024 28803728 PMC5591072

[B37] Xu J. Jin Y. Liu Y. Zhou H. Wang Y. (2019). ORIENT-16: Sintilimab plus XELOX vs placebo plus XELOX as 1st line treatment for unresectable advanced gastric and GEJ adenocarcinoma. Cancer Res. 79, CT213. AACR Annual Meeting:Abstract CT213. 10.1158/1538-7445.AM2019-CT213

[B38] Yu G. Wang L. G. Han Y. He Q. Y. (2012). clusterProfiler: an R package for comparing biological themes among gene clusters. OMICS 16 (5), 284–287. 10.1089/omi.2011.0118 22455463 PMC3339379

[B39] Yu Z. Li Z. Wang C. Pan T. Chang X. Wang X. (2019). Oncostatin M receptor, positively regulated by SP1, promotes gastric cancer growth and metastasis upon treatment with Oncostatin M. Gastric Cancer 22 (5), 955–966. 10.1007/s10120-019-00934-y 30778797

[B40] Zhang X. Zhang P. Shao M. Zang X. Zhang J. Mao F. (2018). SALL4 activates TGF-β/SMAD signaling pathway to induce EMT and promote gastric cancer metastasis. Cancer Manag. Res. 10, 4459–4470. 10.2147/CMAR.S177373 30349378 PMC6188178

[B41] Zhang Y. Liu T. Hu X. Wang M. Wang J. Zou B. (2021). CellCall: Integrating paired ligand-receptor and transcription factor activities for cell-cell communication. Nucleic Acids Res. 49 (15), 8520–8534. 10.1093/nar/gkab638 34331449 PMC8421219

