# Supplementary material for: Circulating neutrophils activated by cancer cells and M2 macrophages promote gastric cancer progression during PD-1 antibody-based immunotherapy
Source: Front Mol Biosci. 2023 Jun 1;10:1081762. doi: 10.3389/fmolb.2023.1081762 (PMC10269372; doi:10.3389/fmolb.2023.1081762)
Supplement: Supplementary file 4 [file Table1.DOCX]

**Supplementary Table1 Clinicopathological characteristics of 88 gastric cancer patients**

| Clinicopathological characteristics | | N | percentage |
| --- | --- | --- | --- |
| Gender | Male | 55 | 62.5 |
|  | Female | 33 | 37.5 |
| Age | <60 | 23 | 26.1 |
|  | ≥60 | 65 | 73.9 |
| Tumor positions | Cardia | 16 | 18.2 |
|  | Corpus | 39 | 44.3 |
|  | Antrum | 33 | 37.5 |
| Borrmann typing | I | 6 | 6.8 |
|  | II | 21 | 23.9 |
|  | III | 50 | 56.8 |
|  | IV | 11 | 12.5 |
| T stage | T1 | 1 | 1.1 |
|  | T2 | 14 | 15.9 |
|  | T3 | 56 | 63.6 |
|  | T4 | 17 | 19.3 |
| N stage | N0 | 18 | 20.5 |
|  | N1 | 11 | 12.5 |
|  | N2 | 23 | 26.1 |
|  | N3 | 36 | 40.9 |
| TNM staging | I | 7 | 8.0 |
|  | II | 25 | 28.4 |
|  | III | 56 | 63.6 |
