# Supplementary material for: Circulating neutrophils activated by cancer cells and M2 macrophages promote gastric cancer progression during PD-1 antibody-based immunotherapy
Source: Front Mol Biosci. 2023 Jun 1;10:1081762. doi: 10.3389/fmolb.2023.1081762 (PMC10269372; doi:10.3389/fmolb.2023.1081762)
Supplement: Supplementary file 10 [file DataSheet1.DOCX]

**Supplementary**

**Figure S1. Clinical information of patients and proportion of cellular types in each sample.**

A: Images of CT scan of enrolled patients; B: Cellular types of collected samples; C: Molecular markers of major cellular types.


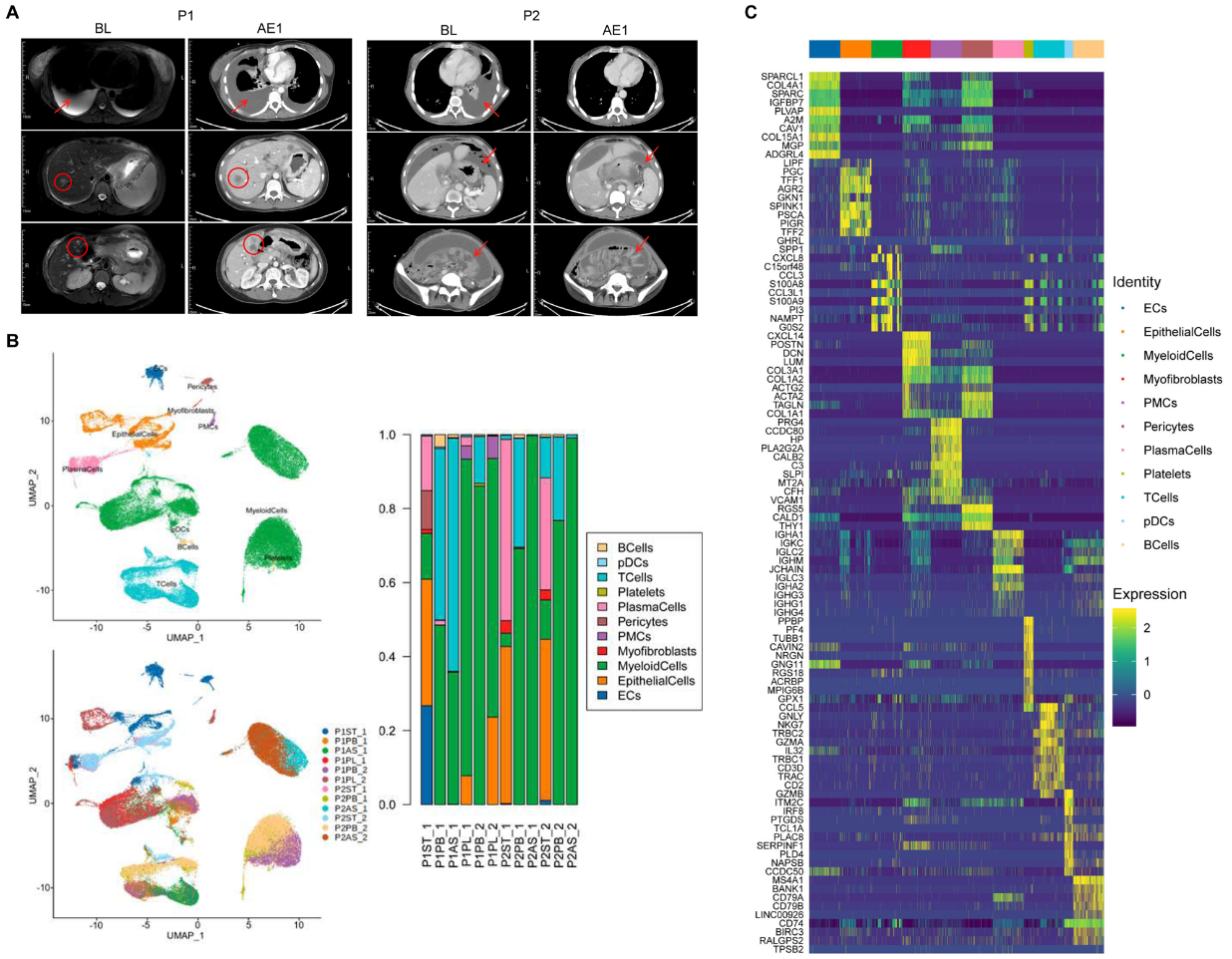


**Figure S2. Cellular subcluster analysis of malignant epithelial cells.**

A: Copy-number variation (CNV) analysis to identified malignant epithelial cells. B: Molecular markers of subclusters of malignant epithelial cells.


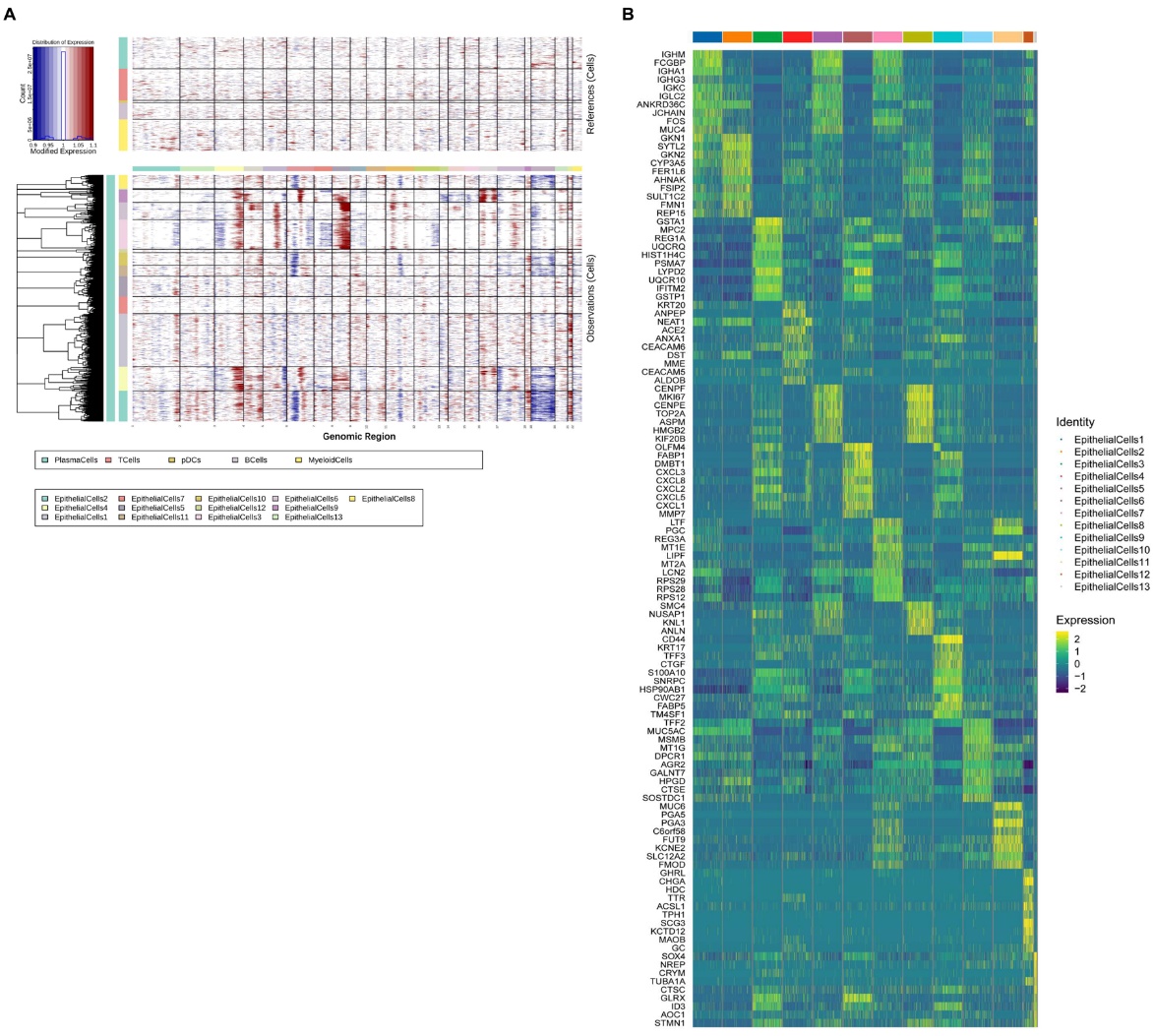


**Figure S3. Cellular interaction analysis**

Heatmap of paired ligands and receptors among subclusters of neutrophil, malignant epithelial cell and M2 macrophage in patient 1 (A) and patient 2 (B).


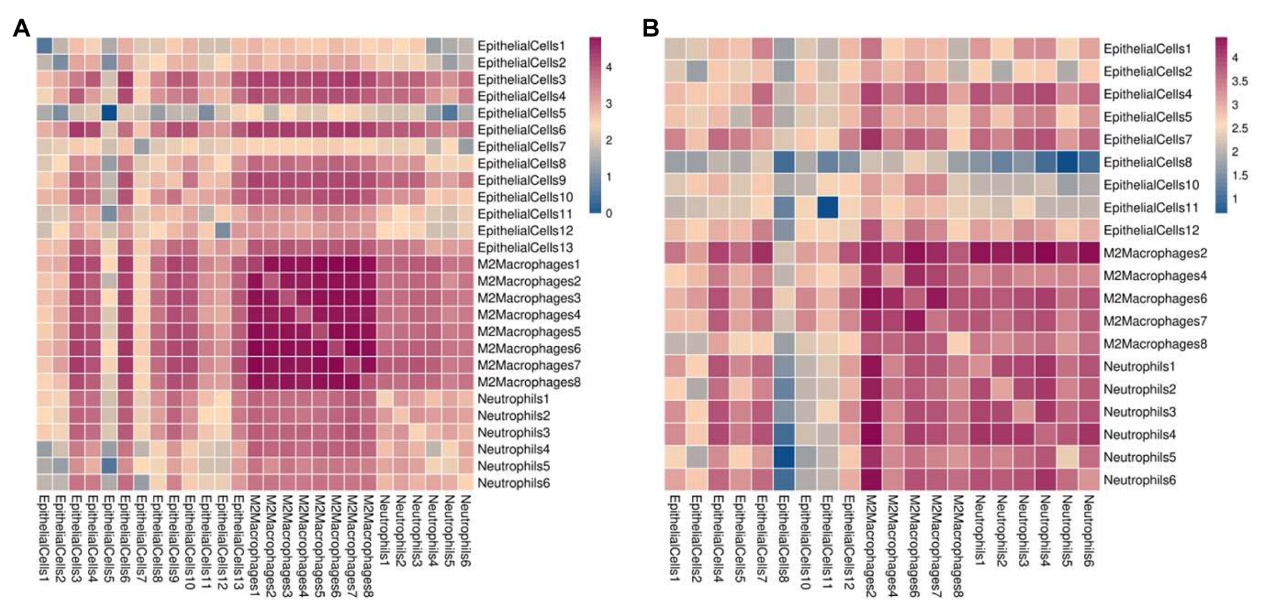


Table S1. Clinicopathological characteristics of 88 gastric cancer patients

Table S2. Number of cells detected in 12 samples.

Table S3. Table S2. Number of myeloid cells detected in 12 samples.

Table S4. Cell number of neutrophil subclusters.

Table S5. List of DEGs of neutrophil subcluster 1 (NE-1).

Table S6. Cell number of malignant epithelial cell subclusters.

Table S7. List of DEGs of malignant epithelial cell subcluster 4 (EP-4).

Table S8. Cell number of M2 macrophage subclusters.

Table S9. List of DEGs of M2 macrophage subcluster 1 (M2-1).

Table S10. List of DEGs of M2 macrophage subcluster 2 (M2-2).
